# The Radical Treatment of Polypoid Disease of the Nose

**Published:** 1902-03-15

**Authors:** 


### THE RADICAL TREATMENT OF POLYPOID DISEASE OF THE NOSE.

Nasal polypi occur in a variety of forms, sometimes a single polyp of large or small dimensions arises without any associated suppurative discharge. Such cases are probably due to some undetermined interference with the vascular structures of the mucosa, with consequent œdematous infiltration of the overlying submucous tissue. In course of time, from the accumulation of fluid, the polyp projects from the surface of the mucous membrane, and, perhaps partly owing to the force of gravity, the outgrowth assumes a pedunculated shape, and this, with the increasing size of the tumour, assumes the common form of a nasal polyp. Such growths may be removed by snare or otherwise, the base cauterised, and the patient permanently relieved.

But when with suppuration of the ethmoidal cells, and various pathological changes in the lining mucosa and the bony walls, numerous polypi are formed, the outlook is very different. The removal of the polypi give temporary relief to the nasal obstruction, but the relief is transient only, for again and again, so long as the suppurative discharge persists, the polypi continue to recur. Moreover, the disease almost always tends to spread by continuity to adjacent nasal accessory cavities. The ultimate result is one of grave danger, not only to the health, but to the life of the patient, more especially when the frontal or the sphenoidal sinus become implicated. Hence it is a matter of very considerable moment that the common multiple nasal polypi with ethmoidal cell disease should receive greater care and more radical treatment than the simpler varieties already alluded to. For the severer and graver form it is not sufficient to remove the polypi and leave the underlying condition to run its course. Hence the method of radical treatment by curettement of the diseased areas becomes a very necessary procedure in many cases. It is often a difficult, and sometimes a dangerous operation, and requires an expert hand; but with due precaution and experience in the treatment of intranasal disease it is generally possible to obtain a lasting cure. Needless to say, it is necessary to recognise and to treat by corresponding radical methods suppuration emanating from the various accessory sinuses which may be concurrent. But we venture to think that hitherto the desirability of radical methods is not sufficiently appreciated by some practitioners, and while milder methods are not to be decried for many polypus cases, the possibility of newer methods offering a better chance of complete and permanent recovery should have due consideration.

